# A smart IoMT based architecture for E-healthcare patient monitoring system using artificial intelligence algorithms

**DOI:** 10.3389/fphys.2023.1125952

**Published:** 2023-01-30

**Authors:** Ahila A, Fadl Dahan, Roobaea Alroobaea, Wael. Y. Alghamdi, Mustafa Khaja Mohammed, Fahima Hajjej, Deema mohammed alsekait, Kaamran Raahemifar

**Affiliations:** ^1^ Indian Institute of Technology, Madras, Chennai, India; ^2^ Department of Management Information Systems, College of Business Administration-Hawat Bani Tamim, Prince Sattam Bin Abdulaziz University, Al-Kharj, Saudi Arabia; ^3^ Department of Computer Sciences, Faculty of Computing and Information Technology-Al-Turbah, Taiz University, Taiz, Yemen; ^4^ Department of Computer Science, College of Computers and Information Technology, Taif University, Taif, Saudi Arabia; ^5^ King Saud University Riyadh, Riyadh, Saudi Arabia; ^6^ Department of Information Systems, College of Computer and Information Sciences, Princess Nourah Bint Abdulrahman University, Riyadh, Saudi Arabia; ^7^ Department of Computer Science and Information Technology, Applied College, Princess Nourah Bint Abdulrahman University, Riyadh, Saudi Arabia; ^8^ College of Information Sciences and Technology, Data Science and Artificial Intelligence Program, Penn State University, State College, PA, United States; ^9^ School of Optometry and Vision Science, Faculty of Science, University of Waterloo, Waterloo, ON, Canada; ^10^ Faculty of Engineering, University of Waterloo, Waterloo, ON, Canada

**Keywords:** cloud computing, healthcare data, internet of medical things (IoMT), high dimensional LDA, multi-objective CSA, hybrid ResNet 18 and GoogLeNet classifier, sensed device

## Abstract

Generally, cloud computing is integrated with wireless sensor network to enable the monitoring systems and it improves the quality of service. The sensed patient data are monitored with biosensors without considering the patient datatype and this minimizes the work of hospitals and physicians. Wearable sensor devices and the Internet of Medical Things (IoMT) have changed the health service, resulting in faster monitoring, prediction, diagnosis, and treatment. Nevertheless, there have been difficulties that need to be resolved by the use of AI methods. The primary goal of this study is to introduce an AI-powered, IoMT telemedicine infrastructure for E-healthcare. In this paper, initially the data collection from the patient body is made using the sensed devices and the information are transmitted through the gateway/Wi-Fi and is stored in IoMT cloud repository. The stored information is then acquired, preprocessed to refine the collected data. The features from preprocessed data are extracted by means of high dimensional Linear Discriminant analysis (LDA) and the best optimal features are selected using reconfigured multi-objective cuckoo search algorithm (CSA). The prediction of abnormal/normal data is made by using Hybrid ResNet 18 and GoogleNet classifier (HRGC). The decision is then made whether to send alert to hospitals/healthcare personnel or not. If the expected results are satisfactory, the participant information is saved in the internet for later use. At last, the performance analysis is carried so as to validate the efficiency of proposed mechanism.

## 1 Introduction

As chronic diseases turn out be vast spread, diagnosis, treatment and monitoring of the diseases are essential. Recently, with the advancements in wearable sensor devices and communication protocol, healthcare system has been supported and enriched. Outlined the method of RPM (Remote patient monitoring) which applies artificial intelligence for its implementation. Data mining, decision support system, cloud computing, Internet of Things and Wearable sensors are reviewed for its challenges, advantages, and applications (El-Rashidy et al., 2021). To extract the information of patients, preprocessing methods are used in techniques of data mining. As data mining techniques are outdated because of its storage and speed issues, big data plays an important role in storing vast volumes of patient information with the aid of storage mechanisms like HDFS and HBase. Development based on the practice-based view was enabled with bigdata analytics transformation model which reveals the capabilities of big data analytics. The model offers a view of big data in healthcare setting strategically. Identification of path-to-value chains for healthcare organizations have also been provided (Ramesh and Santhi, 2020) and (Wang et al., 2018a). Remote patient monitoring system with wearable sensors that uses IoT analytical platform is designed for cardiovascular diseases has been proposed. It can be utilized to check and monitor the current and previous health history of the patient by means of IoT even though the patient is not at the hospital. This method implements compact RPM at low cost with various wearables sensors. Data analytics platform used in this method for monitoring is ThingSpeak (Han et al., 2021). This paper presents an adaptive framework for diabetic patients monitoring that makes use of machine learning methods. Smart devices, smartphones and sensors are the elements used to gather measurements from human body. The patient data collected from the intelligent system further undergoes data classification using machine learning algorithms. The sequential minimal optimization algorithm used provides higher accuracy, precision and sensitivity (Rghioui et al., 2020). An approach named iCloud Assisted Intensive Deep Learning was introduced to provide healthcare medium. This system collects the existing records of health of patients from data repository. Training and test phase of data are performed which would be operated with intensive deep learning principles. The results of the processing are kept in the cloud repositry by IoT enabled features. The integration of IoT with machine learning processes with the smart medical gadgets is used here. Medication monitoring system is devised that utilizes the IoT and deep learning methods to prevent the sensing errors of sensor devices and enhance user experiences by identifying different activities of patients. The method captures images of patients through Open Pose to verify the medication conditions. Reliable communication between medical professional and systems was done through MQTT protocol by periodic transmissions of data (Kondaka et al., 2022) and (Roh et al., 2021). To better estimate health outcomes and improve the effectiveness of smart healthcare services for monitoring health, a Hybrid deep learning techniques was presented. Risk detection was the major concern of this system and hence it increases the efficiency of the healthcare industry. Interpretation of deep learning techniques with IoT and soft sensors helps to identify the concealed patterns. Framework of E-Healthcare monitoring system associated with machine learning techniques was designed as an advanced automation system. As this work is based on IoT applications the monitoring and decision making was performed effectively in regular and periodic manner with proper diagnosis (Rajawat et al., 2021) and (Godi et al., 2020). Additionally, we are concerned for the welfare of our elderly and disabled neighbours when we leave them at home alone for an extended amount of time while going about our daily business. Therefore, it is increasingly important to integrate technology like wearables and healthcare sensors with our healthcare systems to create a more secure and convenient living environment for everyone.

The remaining portion of the manuscript is systematized as follows: Section 2 offers a detailed description on various existing techniques employed beforehand. Section 3 is the depiction of proposed methodology. Section 4 illustrates the performance analysis of proposed work and its comparison with traditional methods. Section 5 narrates the overall conclusion of workflow.

## 2 Related works

In this section various articles of data analytics of patient monitoring and deep learning networks along with Internet of Things are reviewed.

(Harb et al., 2020) this research offered a method for avoiding vulnerable attacks and protect the information of patient on multiple information servers. In comparison with wired sensor networks, remote sensor networks are more vulnerable. The main focus of this paper is to broadcast the patient information through several information servers by using crypto systems in order with a view to preserve the confidentiality of patient information. Patient data stored in sensor networks is safer than ever before.

(Li et al., 2021) provided the specifies of an AI-based system for analyzing large healthcare datasets. As the applications of ML-based methods were used for several IoT applications recently, the healthcare tasks can be processed with IoT applicable sensor devices. Further, advantages and limitations of the existing methods along with some research challenges were presented. Business intelligence that use machine learning methods have recently emerged, giving healthcare practioners and government focused organizations a tool with which to stay completely prepared. Problems like resource allocation, traffic engineering, security and routing can be sorted out by application of ML based techniques.

(Sharma et al., 2021a) provided a framework covid-affected patients may be remotedly monitored. For early covid detection, the model offered was an Internet of Things-based,remotely accessible, alert system bio-wearable sensor system. Biomedical signals like PPG,ECG,accelerometer and temperature were extracted from bio wearable sensor devices in this method. The challenges that encompass the privacy issues and security system were analyzed by the proposed ontology remote monitoring method. The results observed from the simulations shows the efficacy of the model is high with better accuracy.

(Ani et al., 2017) suggested a cerebro vascular treatment monitoring device. By this method of monitoring, future recurrence could be minimized by providing alert to the doctors. With the applications of IoT, data analytics and decision making on real time by health constraints of the patient the medical professional can diagnose the consequences. Thereby preventive treatment can be provided for the patient to safeguard. For diagnosis and treatment, classification algorithms were utilized in this work. Tree based classification-Random Forest method delivered an accuracy of 93 percent in this proposed scheme.

(Rajan Jeyaraj and Nadar, 2019) focused on framework of novel IoT application oriented physiological signal monitoring system for e-healthcare system. Deep neural network-based system was employed for accurate signal prediction and used estimation algorithm in addition. Signal measurement was accomplished by intelligent sensor and myRIO instrument was used for data acquisition. The proposed model also had designed smart monitor for consumer. The model was validated for four physiological signal accuracies with two users. As per results, the proposed automated system has obtained higher accuracy of signal prediction and proved it to be reliable.

(Banerjee et al., 2020) Using concrete examples, you just learned about the fundamentals of big data analytics, the Internet of Things(IoT), and a hybrid type of big data that incorporates machine learning techniques and certain kinds of artificial intelligence to create structured data for remote diagnostics. The development of telemedicine and its integration with Artificial Intelligence to create structured data for remote diagnostics. The development of telemedicine and its integration with artificial intelligence in healthcare delivery was also explored. Importance of Internet of Robotic things and Advancements of telerobotic surgery have been briefly described. Further, wearable devices that targets the biomedical field evolution and healthcare applications were analyzed with standard protocols of machine learning for the system of healthcare.

(Mishra et al., 2020) big data analytics as well as IoT were recently highlighted as emerging fields related to medical fields and also analyzed the system of healthcare monitoring. The model integrates IoT and big data analytics and hence patient information was stored in the cloud. The model developed for implementation could monitor the patient’s health status and the model prediction was faster. It can be implemented at the real time for monitoring health of patient. Thereby medical experts can provide appropriate treatment in advance by earlier predictions.

(Syed et al., 2019) issues in health as well as big data analytics were examined as part of expanding field of study into creating an intelligent healthcare system. The study. s overarching objective is to provide healthcare services to both sick and healthy individuals through remote monitoring using intelligent algorithms, methodologies, and instruments. The approach utilizes sensors worn on the chest, ankle, and wrist to track a person’s everyday activities. The health status of patients may be assessed by the transmission of data from sensors attached towards the internal organs to the caregiver or doctor. At the receiving end, these signals are recorded and processed using big data analytics and machine learning methods. Alzheimer’s sufferers, the elderly, and the overweight may all benefit from remote monitoring using this device. The suggested system was proved to be more accurate in simulations.

(Wang et al., 2018b) examined on the big data analytics based on historical, architectural design and functionalities of components. The capabilities of big data analytics were identified as unstructured data analytical capability, analytical capability for patterns of care, predictive capability, traceability and decision support capability. Also benefits of big data analytics were mapped in terms of operational, organizational, IT infrastructure, strategic and managerial areas. The adoption and support of big data analytics in healthcare organizations were also discussed.

(Pandey and Prabha, 2020) provided the strategy of predicting potential outcomes in the ailment of cardiovascular patients. This analyzes and predicts the malady of cardiovascular diseases with the aid of watch pulse sensors. The reading of sensor was perused and datasets are grouped with therapeutic parameters. By the classification and calculations of artificial intelligence the work is initiated. From the dataset, the extracted information was handled with machine learning techniques. Specifically, Random backwoods classifier algorithm and Decision Tree algorithm were utilized in this work. For detecting heart diseases, Support vector machine was observed as it provides higher accurate outcome with exactness. Hence a framework of foreseeing prior heart disease was demonstrated for earlier prediction with mass screening system.

(Sarmah, 2020) presented a method for monitoring cardiac patients that makes use of Deep Learning modified neural network to aid in the detection and treatment of illness. Authentication, encryption, and categorization are the three components of this suggetsed method. In the first stage, the patient was verified using a substitution cipher and SHA-512. The patient was fitted with an Internet of Things(IoT) sensor wearable device, which was then affixed to his or her body to send the detected data, which was encrypted and transferred anonymously using PDH-AES protocols. DLMNN classifier was used to do classification once the digital information was decrypted. As a result, the data was divided into normal and abnormal categories, and an alarm message was sent to the doctor so that the patient could be treated. Results are confirmed and compared.

(Qureshi et al., 2022) presented the methods of self-monitoring wearable systems, mHealth and telemedicine strategy encourages people with cardiovascular illness to use wearable self monitoring technologies to cut down on hospitalizations. The incorporation of cutting edge methods like computer vision, learning techniques, and cloud services along with diagnostic systems were also discussed. Strengths and limitations of IoT applications in medical services were also identified. By this strategy the dependencies of patients on caregivers could be reduced.

(Tuli et al., 2020) health fog, a framework for integrating deep learning ensembles in to edge computing was devised and used to diagnose cardiac conditions in patients. Since the monitoring a programs need massive amount of data, fog and machine learning were made available in order to offer a novel answer. The term “healthcare fog” refers to the provision of healthcare through Internet of Things(IoT) devices and the administration of patient information.

(Poongodi et al., 2022a) In this research, we suggest a way to automatically suggest a suitable title and add a particular sound to the image. To get this result, two models that have been intensively trained together. The headlines are generated using a combination of natural language processing and cutting-edge computer vision models, and sounds are suggested based on the photographic scene.

(Hamdi et al., 2022) A new and improved CAD system based on a convolutional neural network (CNN) is created that can distinguish between people with normal cognitive function and those suffering from Alzheimer’s disease in order to prevent having a significant negative impact on how well earlier strategies are recognised. T the proposed approach is evaluated and the results indicated that the proposed CAD system provides an accuracy of 96.

(Poongodi et al., 2022b) To diagnose COVID-19, we presented a hybrid deep learning approach with layered technique is employed to assess the severity of the patients’ symptoms and determine whether they are positive for COVID-19 by examining the patient image data. In order to detect the coronavirus quickly and reliably in the lab, a deep learning model using the RNN and CNN algorithms is used to develop a coronavirus rapid test (Dhiman et al., 2022). Citrus fruits that have been labelled with the assistance of a domain expert with four severity levels (high, medium, low, and healthy) serve as the training data for the proposed deep neural network (DNN) model with VGGNet, which is trained to detect targeted parts of the disease with its severity degree.

(Poongodi et al., 2021a) This research assessed upcoming and prospective innovations that will enhance COVID-19 impacts for the general population. This can be done by applying artificial intelligence techniques such as Natural Language Processing (NLP), Machine Learning (ML), and Computer vision to a variety of processing files. This article intends to provide different sets of technologies that can be used to both get rid of COVID-19 and provide information for future generations.

(Kumar et al., 2022) This paper offers a preliminary and unavoidably partial look into the potential benefits of artificial intelligence in the fight against COVID-19 as well as its current difficulties. A number of AI applications, including as early warnings and alarms, monitoring and prediction, data dashboards, diagnosis and prognosis, medications and cures, and social control, may be useful in the battle against COVID-19. AI has the potential to be a very powerful tool in the battle against the COVID-19 virus.

(Bourouis et al., 2022) The computer-aided diagnosis method for identifying abnormalities in breast ultrasound pictures created by combining a wavelet neural network (WNN) with a grey wolf optimization (GWO) algorithm is the main innovation of this work. By calculating the confusion matrix and receiver operating characteristic (ROC) curve, the effectiveness of the suggested methodology is assessed. (Lilhore et al., 2022), (Poongodi et al., 2021b)

The suggested model was deployed using an IoMT-enabled cloud architecture, and its performance was verified using a variety of parameters, including those pertaining to throughput, energy consumption, precision,delay, processing time, and oscillation. As Healthcare IoMT could be configured in various modes of operation the best QoS or accuracy prediction was obtained as required based on requirements of users.

## 3 Proposed work

**FIGURE 1:**
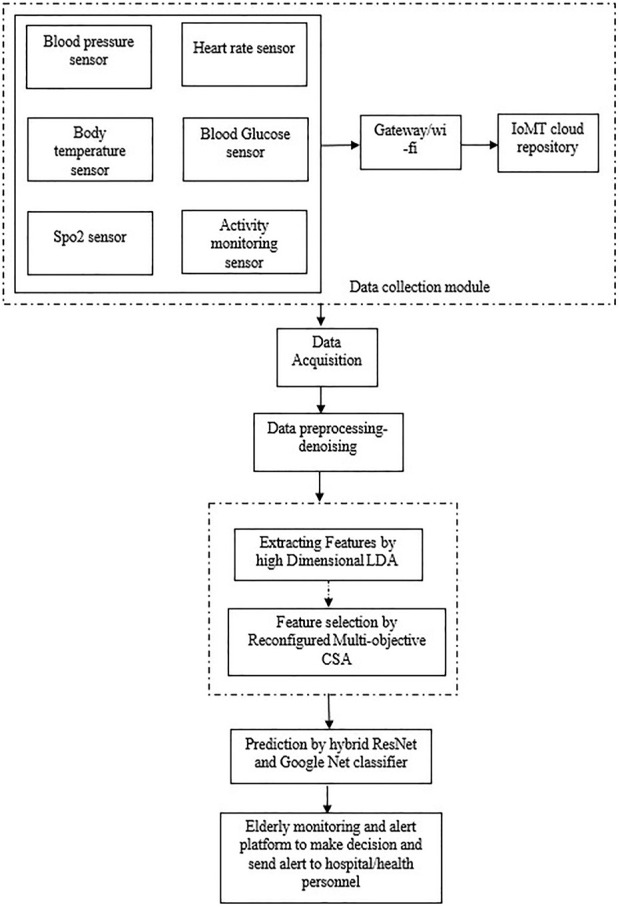
Flow of proposed methodology.

Here, we provide a high level overview of the methods that will be used to implement the plan. Diagram depicting the suggested method’s Figure 1. Cloud system acts as interconnection between the hospital and patients. In real time by the aid of wireless sensor network, smartphones and other digital devices can deliver the updates about patient’s health condition and risks through cloud network. If the patient’s condition is determined to be substandard as compared to the readings provided in lookup table, an automatic alert message will be generated to the physician that was stored in cloud system.

There are two sides of system in the cloud network that are server side and client side that includes various functions like online diagnosis, data querying and uploading patient data. In the server side, the web page is organised on the cloud whereas in the client side, intellectual terminals like medical devices and smart phones are utilized. In this system, the patient could login the web page and query about the status of health for precise period. Hence, the internet technology is integrated to the client for enhanced evaluation and medical data assessment.

### 3.1 Data collection unit

The data from the patient body is collected using sensors such as those used to determine blood pressure, heart beat, temperature and blood glucose, Spo2 sensor, and activity monitoring sensor. The connection between various entities is ensured in IHMS network. There two ranges of communication in network unit called short range and medium range. In these, short range are the protocol technologies utilized within body area network and objects of limited range whereas the medium range supports communication in between the body area network and a base station. In healthcare IoMT mostly preferred communication technology is short range. Various sensor inputs are provided through the data collection unit with the support of some of the communication method Wi-Fi. In general, Wi-Fi network forms quick and easy way of communication in hospitals. Also, for wide range applications Wi-Fi offers compatibility in android phones therefore it aids safety and control. IoMTs cloud archive houses all of the information gathered so far. For the study, 50 patients between the ages of 50 and 70 were examined, and actual health information was acquired from the Chennai Rajiv Gandhi Hospital’s database.

#### 3.1.1 Collection detection unit

The monitoring and detection model of patient are structured to verify the anomalous or abnormal conditions of patient. As the data collection of essential signs like Respiration rate, Heart Rate, Body temperature, Oxygen Saturations and Systolic blood pressure are measured and collected for patient database, it is assigned that set *C* of *p* patients are represented as *C* = [*C*_1_, *C*_2_, *C*_3_, …, *C*_*p*_] where every patient belongs to set *C*. Then it assigned to several biosensor types for collecting the set of vital signs as *S* = [*RR*, *HT*, *BT*, *OS*, *SBP*, …] of the patient data. Let us assume the individual biosensor as $B_{S}^{C}$ for patient *C* and monitors identical vital sign as *s*, each value predicted by biosensors are sent to the record and it is denoted as $R_{S}^{C}=\left[f_{1},f_{2},f_{3},\ldots ,f_{p}\right]$ in the form of vector.

### 3.2 Data acquisition and pre-processing

The input data of the proposed model includes sensing data, medical reports and network data. In remote patient monitoring system, sensors play an important role in connecting the physical world and the patient. Wearable body Sensors can aggregate the patient’s health data and symptoms like ECG, EEG, temperature, oxygen level, BP, sugar level and heart rate. These data are transmitted through wireless standards by maintaining data privacy and security.

In general, these data have inconsistencies, noise, missing values, varied size and formats with higher dimensionality. Since this will produce the output of lower quality it is needed to pre-process the input acquired. Hence pre-processing of sensor data and medical reports are performed initially.

• **Sensor data pre-analysis:** The normal and abnormal conditions of various attributes like BP, sugar level, heart rate etc. are analysed for the patient by sensors along with body mass index, gender, age and activities. In the first step, for easy parsing, the dataset is transformed to comma separated value files. Then names are specified for each attribute and presented with the numerical values. In the second step, identities of sensor data are assigned. These generated features deliver the final dataset of patient and loaded to the cloud processor.
• **Pre-processing data input:** The sensor data are filtered to eliminate the noise and inconsistencies with the use of Kalman filtering and unsupervised filter is utilised in our approach to replace the missing values presented in the dataset of available data with average mean and modes. Unused attributes are eliminated with the support of unsupervised filter to get the maximum variance attribute. The obtained numerical values are normalized by the normalization method to limit the range between 0 and one for further processing and classification. By the application of EmEditor tool the dataset is divided into n data files which are loaded into the cloud processor. Table 1 shows the sample dataset.

**TABLE 1:** sample dataset.

Person age pulserate temp SPO2
01 54 76 38 93
02 55 73 40 92
03 64 72 45 91
04 68 74 50 94

### 3.3 Feature extraction by high dimensional Linear Discriminant analysis

During the process of extraction of features, multidimensional reduction is done along with searching the directions for maximum class separation. Different data classes are separated by High Dimensional Linear Discriminant Analysis method. The data is classified and diminished for specific dimension. The HDLDA’s covariance class matrix was featured by,

$$C_{r}=\overset{n}{\underset{n=1}{\sum }}C_{n}$$ (1)

Here,

$$C_{n}=\underset{k\epsilon S_{n}}{\sum }\left(y_{k}-x_{n}\right){\left(y_{k}-x_{n}\right)}^{M}$$ (2)

$$x_{n}=\frac{1}{T_{n}}\underset{k\epsilon S_{n}}{\sum }\left(y_{k}\right)$$ (3)

In class *S*_*n*_, the count of total patterns is *T*_*n*_. Additionally, *y*_*k*_ is the discrete wavelet transform coefficient vector with pattern *k*, then the covariance matrix among the class is featured as,

$$C_{B}=\overset{n}{\underset{n=1}{\sum }}T_{n}\left(x_{n}-x\right){\left(x_{n}-x\right)}^{M}$$ (4)

$$x=\frac{1}{T}\overset{k}{\underset{k=1}{\sum }}y_{k}=\frac{1}{T}\overset{k}{\underset{k=1}{\sum }}T_{n}x_{n}$$ (5)

Here *x* is the global average mean of the data and the total covariance matrix is denoted as

$$C_{M}=C_{B}+C_{R}$$ (6)

The calculation of projection matrix is shown as,

$$R=\arg_{R}\left(\max{\left(RC_{R}R^{N}\right)}^{-1}\left(RC_{B}R^{N}\right)\right)$$ (7)

The coefficients of LDA are got from the projection matrix as,

$$m=R^{N}y$$ (8)

Here, the DWT coefficient vector is denoted as y and m is the vector coefficient of LDA.

### 3.4 Feature selection by reconfigured multi-objective cuckoo search Algorithm

The process of feature selection is implemented by Reconfigured Multi-objective Cuckoo Search Algorithm in our proposed system to select the best extracted features so as to obtain output of higher efficiency. Reconfigured Multi-objective Cuckoo Search (RMCS) Algorithm is a metaheuristic optimization technique that is, inspired by the biological behavior characteristics of cuckoo. Based on two terminologies like randomization and stochastic search the algorithm was developed. At first, the local search space is explored for local optima and expanded further to derive a global optimal solution. By means of modifying the step size and values of parameters, the performance boundary can be personalized dynamically.

The cuckoo has an oblige behavior and it is a brood parasitic organism that hinge on other birds for its reproduction. Cuckoo also depends on other host birds to raise its young ones. The Cuckoo search is initiated by activities of genetic influence, for instance foraging of cuckoo. Generally, in the nest of host birds cuckoo lays its egg and hatch it. These young ones hatched seeks the attention of host for obtaining food. Also, it replicates the exterior host attributes that is, based on the approaches of exploration and exploitation. Algorithm 1

Algorithm 1 Reconfigured Multi-Objective Cuckoo Search.

**Start**
Produce original population of host nest as *n*
**Function objective:** *f*(*y*)
Find eggs of best fit and rank
**while** (*s* > *MaxProduction*) or stop criterion **do**
*S* = *s* + 1
  Obtain a cuckoo arbitrarily or produce new solution by levy flights
  Find best fit or quality *Q*_*i*_
**Select** a nest randomly as *k*
**if** (*Q*_*i*_ > *Q*_*k*_) **then**
**Swap** *k* by new solution
**end if**
  With a probability Pe unfit nest is abandoned and formed new nest
  Assess rank and best fit the solution and accept current as best
**end while**
Send process output and visualization
**End**

A novel solution is derived by the levy flights methods hence. Some of the constraints followed in the algorithm are.

• At a time, one egg will be laid by each cuckoo and leaves it randomly in nest selected.
• The best eggs are grown as the future generation.
• There are fixed number of nests.

But there is a probability that the unknown eggs may be identified by the bird host. In that condition, the host bird will leave its nest or discard the eggs.

Hence the process of feature selection is executed with the RMCS algorithm which is then followed by the process of classification.

### 3.5 Model generation by hybrid ResNet and GoogleNet classifier for classification and alert system

From the features selected, the prediction model is generated with hybrid ResNet and GoogleNet classifier. ResNet classifier is a completely connected neural network that increase in size as the hidden layer neurons and input data dimension raises. Hence this may affect the speed of training model. to get solution to this issue, in our work we have used CNN with features of local connection along with parameter sharing. This can decrease the number of model attributes and step up the speed of training the model. the ResNet classifier model utilized can abstract many features of ECG data and Heart rate data from the similar input data that results in getting internal structure of representation accurately and efficiently. The identification and classification of heartbeat classes based on database are realized with higher precision by the support of ResNet. The attributes determined in ResNet are optimizer, learning rate and the loss function that performs classification qualitatively. Algorithm 2

Algorithm 2 ResNet-18 Classifier.

**Require:** ECG signals
**Ensure:** classification of ECG signals and heartbeat
  Set the learning rate of layer as *α* minimum error as min_*e*_, maximum iteration epoch, full batch
  Determine the value *φ* = *b*_*j*_, *w*_*j*_, *φϵ*(1, 2, …, *M*)
  Produce random weights of ResNet
  ResNet model = Init ResNet model *φ*)
**if** *err* > min_*e*_ and *iter* < *epoch* **then**
**Set** *err* = 0
**for** *batch* = 1 to full batch training **do**
*φ* = *ResNetmodel*.*train* (signals feature, signals training)
**Update** *φ*
*err* = *err* + *mean*(*K*(*φ*))
**end for**
*iterj* = *j* + 1
**end if**
**return** result with lesser *φ*

At last, features of output data are represented from the wholly connected layer to vector of one-dimension.

GoogleNet is the kind of two-stage object detectors in which the features with low-level are aided to identify the object. Also, deep convolution neural network is utilized for classifying the objects located. Hence object location and classification are enhanced by the performance of feature representation. Increasing the network’s number of brain cells and layers enhances the system’s performance. As the size of network is increased, there will be increased parameters. Also, as there is reduction in dimensionality of 1 × 1 convolution kernel the computational complexity is minimized by maximizing the width and depth of the network. Inception modules present in the network can modify the serial form to parallel form. It also replaces the wholly connected layer by the average pooling layer in the GoogleNet. Algorithm 3

Algorithm 3 GoogleNet Classifier.

**Require:** Input Image
**Ensure:** Object Detection and Classification
*d* ← *b*.*shape*
*part* ← *self*.*Nb*
*sqrk* ← *K*.*sqr*(*d*)
*max*_*c*_ *hannel* ← *K*.*alloc*(0, *c*, *cha* + 2*part + *d*)
*qrk* ← *K*.*init*_*s*_ *ubtensor*(*max*_*c*_ *hannel*, *sqrk*)
*range* ← *self*.*l*
*gamma* ← *self*.*gamma*/*self*.*Nb*
**for** *j* in range (*self*.*Nb*) **do**
*range* ← gamma*sqr(*Nb*)
*range* ← range**self.*Nb*
*x* ← *x*/*range*
**end for**
**return** *x*

#### 3.5.1 Alert system

The system of warning is dependent on the vital signs, that is, *C* which can be utilized by the medical professional in the hospital so as to track on the patient’s abnormality condition. Every vital sign from the collected data record $f_{p}\epsilon R_{S}^{C}$ in comparison with normal level is used to calculate score value *S*_*y*_ between 0 and three where 0 indicates the condition is normal and other values show the criticality of the patient with increase in value of score. So, a vector set of record scores $V_{S}^{C}=\left[v_{1},v_{2},v_{3},\ldots ,v_{p}\right]$ is evaluated for every record $R_{S}^{C}$ . In that case, alert system is aided for determining the proper action. If the score value is high the patient needs medical attention as it should be considered as an emergency case and low value of score grants to decrease the patient monitoring frequency. Hence the alert system works based on data network and warns the medical professional.

## 4 Performance analysis

The performance analysis of the proposed model is carried and the outcomes attained are compared with existing methods (Latif et al., 2022), (Khan et al., 2021), (Yang et al., 2017) and (Sharma et al., 2021b) to validate the efficiency of proposed methodology. The outcomes attained from the analysis is projected below in the table and graphical representation.

Table 2 shows that the performance parameters for simlation. The above parameters are recorded for 50 patients: respiration rate (RR), systolic blood pressure (SBP), heart rate (HR), oxygen saturation (OS), and blood sugar level (SL). Readings are gathered every second using biosensor sensors. A file with a log of each patient’s about 500 readings was used in our model. The biosensor sends the data to the caretaker after receiving it from its particular file on a regular basis.

**TABLE 2:** Performance parameters for simulation

Parameters	Symbol	Values	
no of patients	*Γ*	50	
No of features	*Α*	[RR, SBP, HR, OS, SL]	
period size	*Τ*	500 records	
minimum sensing frequency	Fmin	5, 10, 15 percentage of	*γ*
No of time steps	*ɛ*	100,500 records	
Training data size	*Ρ*	1 period	

**FIGURE 2:**
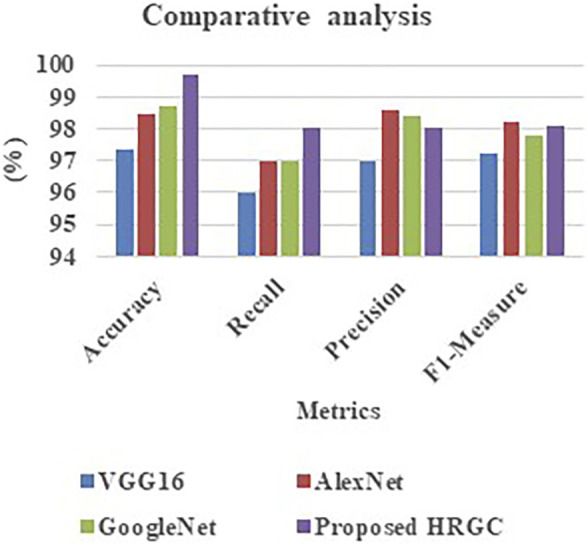
Evaluation of Recall,F1, and other quality measures.

Table 3 and Figure 2 signifies the performance comparison of accuracy, precision, recall, and F1-measure made for the proposed HRGC method and the existing methods like VGG16, AlexNet, and GoogleNet. From the analysis it was evident that the proposed model is better on comparing the traditional techniques.

**TABLE 3:** Performance comparison of accuracy, precision, recall, and F1-measure (Latif et al., 2022).

Technique	Accuracy	Recall	Precision	F1-measure
VGG16	97.32 C 96	97	97.2	
AlexNet	98.49	97	98.6	98.2
GoogleNet	98.71	97	98.4	97.8
Proposed HRGC	99.69	98	98	98.1

Accuracy, precision, recall, F1-measure, and training duration are selected as measures by which the suggested technique may be effectively validated and the improvement of the proposed model proven.

From the outcome, it was observed that the accuracy level ranges from 97.32% to 98.71% in the existing models, whereas the proposed method offers highest range of accuracy 99.69%. Also, the proposed model offers high rate of precision (98%), recall (98%), and F1-measure (98.1%).

**FIGURE 3:**
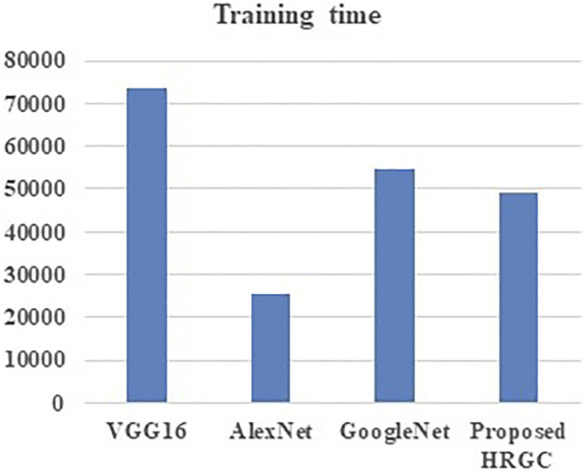
Performance comparison of training time.

Table 4 and Figure 3 signifies the performance comparison of training time made for the proposed HRGC method and the existing methods like VGG16, AlexNet, and GoogleNet. From the analysis it was evident that the proposed model shows reduced training time on comparing the traditional techniques.

**TABLE 4:** Performance comparison of training time (Latif et al., 2022).

Technique	Training time
VGG16	73756
AlexNet	25568
GoogleNet	54866
Proposed HRGC	49201

From the analysis, it was observed that the training time ranges from 25,568 to 54,866 s in case of existing models and about 49201 s in proposed model which is lower than existing ones.

**FIGURE 4:**
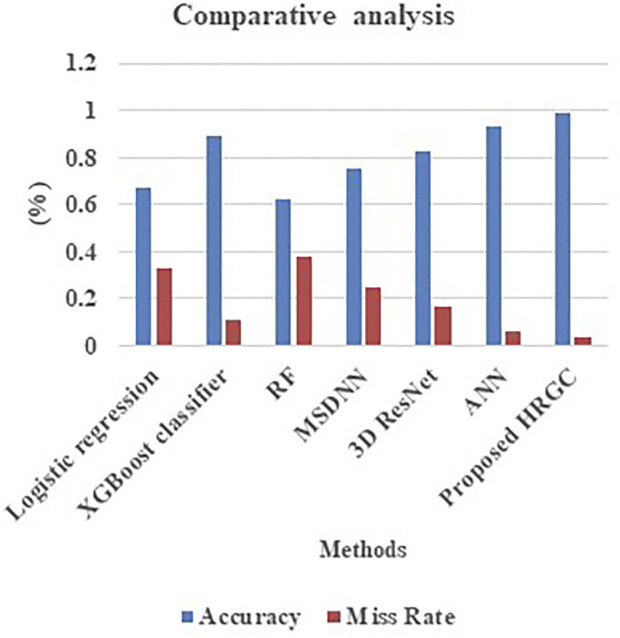
Performance comparison of accuracy and miss rate.

Table 5 and Figure 4 signifies the performance comparison of accuracy and miss rate made for the proposed HRGC method and the existing methods like Logistic regression, XGBoost, RF, MSDNN, 3D Resnet, ANN and proposed HRGC. From the analysis it was evident that the proposed model offers high accuracy rate and lower miss rate on comparing the traditional techniques.

**TABLE 5:** Performance comparison of accuracy and miss rate (Khan et al., 2021).

Technique	Accuracy	Miss rate
Logistic regression	0.672	0.328
XGBoost classifier	0.892	0.108
RF	0.619	0.381
MSDNN	0.754	0.246
3D ResNet	0.830	0.170
ANN	0.936	0.064
Proposed HRGC	0.989	0.034

In table 6, the amount of time needed for computation is evaluated for the feature selection algorithm. The computational time is evaluated and the comparison is made. From the analysis, it was obvious that the proposed method offers lower computational time. Moreover, the fitness of proposed method was superior to other existing methods.

**TABLE 6:** Performance comparison of computation time and fitness time (Yang et al., 2017).

Technique	Computation time (min)	Fitness
NBBA	1.34	1.87
BBA	1.02	1.65
IBPSO	2.01	0.85
BPSO	1.71	0.87
MBAFS	2.84	0.76
Proposed CS	0.81	3.98

**FIGURE 5:**
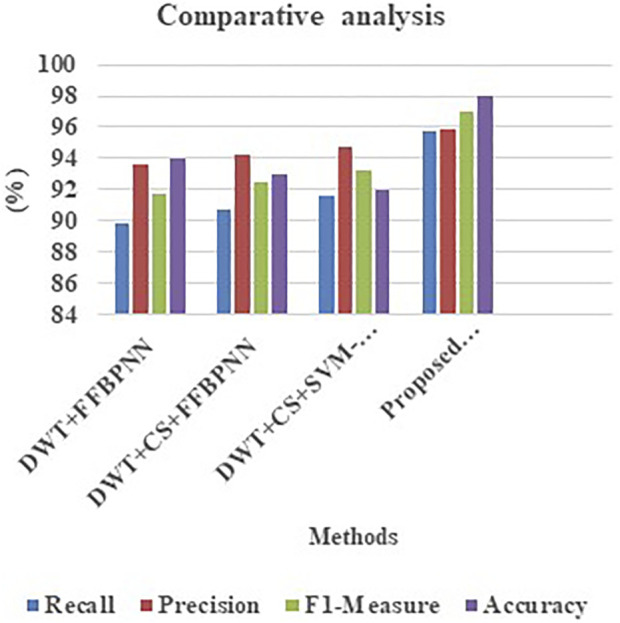
Comparative analysis of recall, precision, F1-measure, and accuracy.

Table 7 and Figure 5 is the comparison made for various existing methods and the proposed model in terms of accuracy, recall, precision, and F1-measure. The proposed variant shows highest performance metrics on comparing other existing variants like combining DWT with FFBPNN, then CS-optimized DWT, and finally SVM-FFBPPNN, DWT optimized by CS, and subsequently HRGC’s suggested LDA.

**TABLE 7:** Comparative analysis of recall, precision, F1-measure, and accuracy (Sharma et al., 2021b).

Technique	Recall	Precision	F1-measure	Accuracy
DWT + FFBPNN	89.8	93.6	91.73	94
DWT + CS + FFBPNN	90.76	94.22	92.46	93
DWT + CS + SVM-FFBPNN	91.65	94.8	93.23	92
Proposed LDA + CSA + HRGC	95.7	95.9	97	98

**FIGURE 6:**
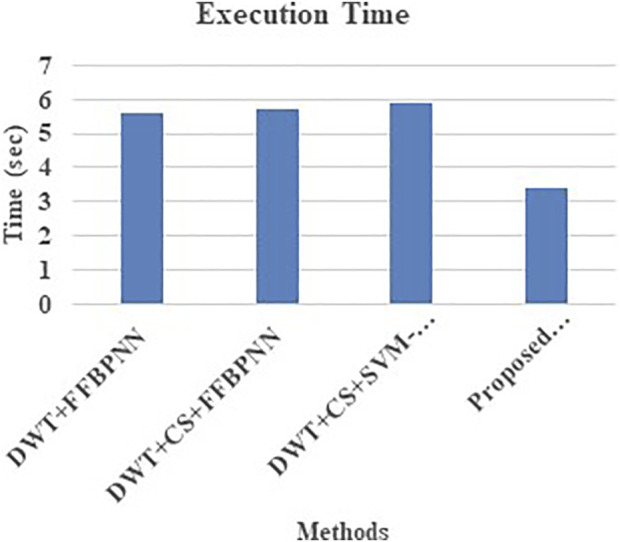
Comparative analysis of Execution time.

Reliability, recall,accuracy, and F1-score are compared across several variations of both the current approaches and the proposed models in Table 8 and Figure 6. The primary goal of analyzing completion time is to catalog the change in runtime that corresponds to each improvement. The effectiveness of the suggested technique is shown by the shorter runtime of the proposed version compared to other variants.

**Table 8:** Comparative analysis of Execution time

Technique	Execution Time
DWT+FFBPNN	5.6
DWT+CS+FFBPNN	5.7
DWT+CS+SVM-FFBPNN	5.9
Proposed LDA+CSA+HRGC	3.4

## 5 Conclusion

At first, the patient data was collected from the patient body using sensed devices and the information were transmitted through gateway/Wi-Fi and is then kept in IoMT cloud repository. The data stored was acquired, preprocessed to denoise the collected data. The features were extracted using high dimensional LDA and the best optimal features are selected using reconfigured Multi-objective CSA. Then, the prediction of abnormal/normal data was made with the use of Hybrid ResNet 18 and GoogleNet classifier. The decision-making system decides whether to send alert or not based on the type of data predicted that is, normal or abnormal one. In case, there occurs some abnormality in the data predicted alter will be send to hospital/healthcare personnel. The performance assessment was made finally in terms of accuracy, miss rate, execution time, computation time, precision, recall, and F1-measure. From the estimation made, the outcomes attained are compared with traditional methods. It was revealed from the analysis that the proposed system functions well on comparing traditional methods. In future, this work can be extended by implementing video based system.

## Data Availability

The original contributions presented in the study are included in the article/supplementary material, further inquiries can be directed to the corresponding author.
